# A Proteomics-Based Analysis Reveals Predictive Biological Patterns in Fabry Disease

**DOI:** 10.3390/jcm9051325

**Published:** 2020-05-02

**Authors:** Abdellah Tebani, Wladimir Mauhin, Lenaig Abily-Donval, Céline Lesueur, Marc G. Berger, Yann Nadjar, Juliette Berger, Oliver Benveniste, Foudil Lamari, Pascal Laforêt, Esther Noel, Stéphane Marret, Olivier Lidove, Soumeya Bekri

**Affiliations:** 1 Department of Metabolic Biochemistry, Rouen University Hospital, 76000 Rouen, France; abdellah.tebani@yahoo.com (A.T.); celine.lesueur@chu-rouen.fr (C.L.); 2 Department of Internal Medicine, Groupe Hospitalier Diaconesses Croix Saint Simon, Paris, France & INSERM U 974, 75014 Paris, France; wmauhin@hopital-dcss.org (W.M.); olidove@hopital-dcss.org (O.L.); 3 Department of Neonatal Pediatrics, Intensive Care and Neuropediatrics, Rouen University Hospital, 76000 Rouen, France; lenaig.donval@gmail.com (L.A.-D.); stephane.marret@chu-rouen.fr (S.M.); 4 Normandie Univ, UNIROUEN, CHU Rouen, INSERM U1245, 76000 Rouen, France; 5 CHU Clermont-Ferrand, Hopital Estaing, CRB-Auvergne, 63003 Clermont-Ferrand, France; mberger@chu-clermontferrand.fr (M.G.B.); jberger@chu-clermontferrand.fr (J.B.); 6 Université Clermont Auvergne, EA 7453 CHELTER, 63000 Clermont-Ferrand, France; 7 Neurology Department, Reference center for Lysosomal Diseases, Hôpital Pitié-Salpêtrière, 75013 Paris, France; yann.nadjar@aphp.fr; 8 Department of Internal Medicine, Hôpital Pitié-Salpêtrière, Paris, France & INSERM U 974, 75013 Paris, France; olivier.benveniste@aphp.fr; 9 Department of Metabolic Biochemistry, Pitié-Salpêtrière Hospital, APHP-Sorbonne university, 75013 Paris, France; foudil.lamari@aphp.fr; 10 Neurology Department, Hôpital Raymond-Poincaré, AP-HP, 92380 Garches, France; pascal.laforet@aphp.fr; 11 Department of Internal Medicine, Centre Hospitalier Universitaire, 67000 Strasbourg, France; esther.noel@chru-strasbourg.fr

**Keywords:** inborn errors of metabolism, Fabry disease, lysosomal storage diseases, proteomics, systems biology, machine learning

## Abstract

*Background*: Fabry disease (FD) is an X-linked progressive lysosomal disease (LD) due to glycosphingolipid metabolism impairment. Currently, plasmatic globotriaosylsphingosine (LysoGb3) is used for disease diagnosis and monitoring. However, this biomarker is inconstantly increased in mild forms and in some female patients. *Materials and Methods*: We applied a targeted proteomic approach to explore disease-related biological patterns that might explain the disease pathophysiology. Forty proteins, involved mainly in inflammatory and angiogenesis processes, were assessed in 69 plasma samples retrieved from the French Fabry cohort (FFABRY) and from 83 healthy subjects. For predictive performance assessment, we also included other LD samples (Gaucher, Pompe and Niemann Pick C). *Results*: The study yielded four discriminant proteins that include three angiogenesis proteins (fibroblast growth factor 2 (FGF2), vascular endothelial growth factor A (VEGFA), vascular endothelial growth factor C (VEGFC)) and one cytokine interleukin 7 (IL-7). A clear elevation of FGF2 and IL-7 concentrations was observed in FD compared to other LD samples. No correlation was observed between these proteins and globotriaosylsphingosine (LysoGb3). A significant correlation exists between IL-7 and residual enzyme activity in a non-classical phenotype. This highlights the orthogonal biological information yielded by these proteins that might help in stratifying Fabry patients. *Conclusion*: This work highlights the potential of using proteomics approaches in exploring FD and enhancing FD diagnosis and therapeutic monitoring performances.

## 1. Introduction

Fabry disease (FD, OMIM #301500) is an X-linked and progressive inherited metabolic disease characterized by cellular dysfunction and microvascular pathology related to lysosomal glycosphingolipid metabolism impairment [1]. Incidence ranges from one in 40,000 to one in 117,000 in the general population [2]. However, some screening studies suggested higher incidences of FD with a rate of one in 3100 in northwestern Italy [3], one in 3000 in Austria [4], one in 3024 in Japan [5], one in 2913 in the United States of America (USA; Missouri) [6], and one in 1500 in Taiwan [7]. FD is due to an absence or a decrease of the lysosomal α-galactosidase A activity (GalA; EC 3.2.1.22). This leads to a progressive lysosomal accumulation of globotriaosylceramide (Gb3) and related glycosphingolipids such as galabiosylceramide in different cell types, including endothelial and vascular cells, renal cells (podocytes, tubular cells, glomerular endothelial, mesangial, and interstitial cells), cardiac cells, and nerve cells (neurons, Schwann cells). This continuous deposition may lead to significant cellular impairments and organ failures [8]. There is a significant reduction of life expectancy and the main causes of death are related to organ damage mainly, kidney, heart, and the central nervous system [9]. Most Fabry patients remain clinically asymptomatic in early life; however, in the classical FD phenotype, symptoms arise in childhood or adolescence and include neuropathic pain, angiokeratoma, and cornea verticillata [10]. Particularly in classical FD, serious complications developing in adulthood may include progressive renal insufficiency, gastrointestinal dysfunction, cardiac complications (arrhythmia, hypertrophic cardiomyopathy), and/or cerebrovascular complications such as early stroke. Patients may also present vascular ectasia and tortuosity [10]. Males with pathogenic variants may exhibit low to absent residual GalA activity and may undergo the full clinical spectrum of the disease. In heterozygote female, the presentation is more heterogeneous, may depend on the normal/mutant GalA ratio in different tissues [11], and is variant type-dependent [12]. This clinical variability is due to the X-inactivation in female patients and may be increased in male and female patients by the effect of putative modifier genes as demonstrated by the lack of *GLA* genotype–phenotype correlation. The diagnosis of FD in males is often made by characterizing a deficiency in GalA activity in a blood sample (white blood cells) [13], plasma/serum [14], or a dried blood spot [15]. A deficiency in GalA activity can also be demonstrated in lymphoblasts, cultured fibroblasts, tears, or urine [16]. Furthermore, the diagnosis is confirmed by molecular analysis of the *GLA* gene. However, the enzymatic assessment is unreliable for female carriers given the random inactivation of the X-chromosome. In this case, molecular analysis is of great help to detect heterozygous individuals. The concentrations of the storage product, Gb3, in plasma [17,18] or urine [19] are inconsistently increased in the late-onset forms and in female patients. Globotriaosylsphingosine (LysoGb3), the deacylated derivative of Gb3, allows for greater discrimination, but false negatives in very-late-onset forms and some female patients were reported [20,21]. Early diagnosis of FD is very important for better disease management; thus, suitable methods for high-risk population screening were developed to assess GalA activity in dried blood spots [22] and storage products in urine collected on filter paper [23]. Regarding therapeutic strategies, enzyme replacement therapy (ERT) by intravenous exogeneous human α-galactosidase A markedly enhances FD management. Two ERTs are currently available: recombinant (algalsidase β) [24] or gene-activated human α-galactosidase A enzyme [25]. A new therapeutic strategy was developed recently based on the increase of the enzymatic activity of mutated protein using a pharmacological chaperone that can facilitate its proper folding [26]. However, monitoring the effects of specific treatments in a clinical setting is still challenging due the lack of robust surrogate markers of treatment response and the large phenotype and genotype variability in FD [27,28]. All these therapeutic strategies significantly improved the course of the disease and the quality of life of the patients, but they do not completely stop the course of the disease. This suggests that the molecular pathophysiology of Fabry disease is not yet fully understood and there is a real need of more accurate patient stratification for better healthcare management.

It is recognized that FD is underdiagnosed with usually a significant delay between the onset of the first signs and diagnosis [29]. A better understanding of FD biology may enhance our screening and diagnosis tools with potential new biological signatures.

The post-genomic era allowed remarkable advances in omics technologies that led to the generation of a tremendous wealth of information to support different medical fields including inherited metabolic diseases [30,31,32,33]. This omics surge allowed integrative interrogation of complex data streams retrieved from biological systems. This is mainly based on bioinformatics, data modeling, and systems biology strategies. This holistic approach has the potential to promote unbiased, data-driven, and hypothesis-free strategies to study disease states. Moreover, it overcomes the limits of the reductionist aspect of hypothesis-driven approaches [32]. Several proteomics-based studies were previously reported in FD [34,35,36,37,38,39,40,41,42,43]. We describe here a targeted proteomics study aiming to determine underlying proteomic-based biological signatures that could discriminate Fabry patients by comparing them with healthy subjects and with three other LDs including Pompe, Niemann–Pick type C, and Gaucher disease. In addition, we aimed to compare the unveiled proteomic signatures with conventional FD biomarkers.

## 2. Materials and Methods

### 2.1. Patients and Blood Samples

**Figure 1:**
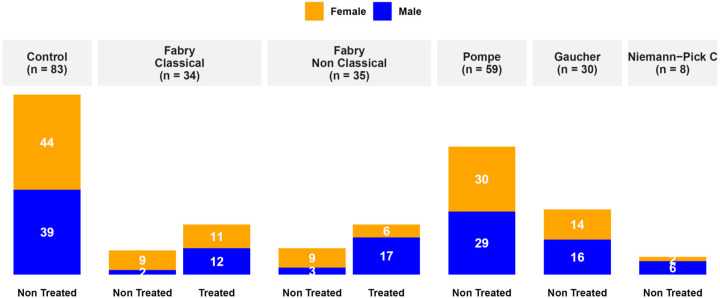
Overview of the cohort.

Blood samples were collected from patients with a confirmed Fabry diagnosis retrieved from the French Fabry cohort (FFABRY) using enzymatic test, genetic test, or both. FFABRY is a French multicenter cohort of patients with an enzymatic and/or genetic diagnosis of FD [44]. Sixty-nine patients were included: 34 with classical phenotype including 20 females (age range: from 20.2 to 75.4 years, mean age: 48.2 years) and 14 males (age range: from 20.2 to 59.4 years, mean age: 38.9 years), 35 with non-classic phenotype including 15 females (age range: 16.7 to 66.3 years, mean age: 45.9 years) and 20 males (age range: 17.1 to 74.2 years, mean age: 48.7 years). Forty-six were treated, 12 with Agalsidase α (nine classical and three non-classical), 21 with Agalsidase β (10 classical and 11 non-classical), one with Migalastat (non-classical), 10 with Agalsidase α and Agalsidase β (four classical and six non-classical), one with Agalsidase α and Migalastat (non-classical), and one with all three, i.e., Agalsidase β, Agalsidase α, and Migalastat (non-classical). The mean cumulative treatment duration time was 6.4 years. Genotyping was done in 63 patients out of 69. Twelve and 25 missense variants were found in classical and non-classical Fabry patients, respectively. For mutations leading to a truncated protein (deletion, frameshift, or non-sense mutations), 17 and nine were found in classical and non-classical Fabry patients, respectively. A summary overview of the clinical characteristics, phenotype, treatment, laboratory investigations, and *GLA* genotype of Fabry patients is presented in Table 1. The full data are presented in Tables S1 and S2 (Supplementary Materials). Human controls were selected with similar mean age and sex with no significant medical conditions. Control plasmas were purchased from Biovit (West Sussex, UK). We analyzed plasma samples from 83 healthy donors, 39 males (ages range: from 20 to 64 years, mean age: 39 years) and 44 females (age range: from 21 to 69 years, mean age: 44 years). To test our findings, we also collected other LD samples including 59 Pompe, 18 Niemann–Pick type C, and 30 Gaucher disease (GD). The overall summary of the cohort is presented in Figure 1. GD Patient samples were taken from the national multi-center GD biological collection (ELODIE-MG, #DC-2012-1652), with the agreement of the French GD expert group (*Comité d’Evaluation du Traitement de la maladie de Gaucher*, *CETG*) as the Scientific Committee. The study was approved by the Institutional Ethics Committee Research (Ethics Board of Rouen University Hospital - CERNI E2016-21).

### 2.2. Targeted Proteomic Analysis

Plasma proteins were measured using electrochemiluminescence assays (Meso Scale Discovery [MSD], Rockville, MD, USA). V-PLEX Human Biomarker 40-Plex Kit including C-reactive protein (CRP), Eotaxin, Eotaxin-3, fibroblast growth factor 2 (FGF2), granulocyte-macrophage colony-stimulating factor (GM-CSF), intercellular adhesion molecule 1 (ICAM-1), interferon gamma (IFN-γ), interleukin 10, interleukin 12/interleukin 23p40, interleukin 12p70, interleukin 13, interleukin 15, interleukin 16, interleukin 17A, interleukin 1α, interleukin 1β, interleukin 2, interleukin 4, interleukin 5, interleukin 6, interleukin 7, interleukin 8, IP-10, Monocyte chemoattractant protein 1 (MCP-1), Monocyte chemoattractant protein 4 (MCP-4), macrophage-derived chemokine (MDC), macrophage inflammatory proteins 1 alpha (MIP-1α), macrophage inflammatory proteins 1 beta (MIP-1β), placental growth factor (PlGF), Serum amyloid A (SAA), thymus and activation regulated chemokine (TARC), Angiopoietin-1 receptor (Tie-2), tumor necrosis factor alpha (TNF-α), tumor necrosis factor beta (TNF-β), vascular cell adhesion protein 1 (VCAM-1), vascular endothelial growth factor (VEGF-A), vascular endothelial growth factor C (VEGF-C), vascular endothelial growth factor D (VEGF-D), and vascular endothelial growth factor receptor 1 (VEGFR-1/Flt-1). Data were acquired using a SECTOR S 6000 plate reader (Meso Scale Diagnostics, Rockville, MD, USA).

### 2.3. Plasma lysoGb3 Analysis

The lysoGb3 concentration was measured as previously described [44]. Plasma samples were analyzed using ultra-performance liquid chromatography coupled to tandem mass spectrometry (UPLC–MS/MS). In glass tubes, ethylenediamine tetraacetic acid (EDTA)/plasma was mixed with glycine/lysoGb3 (100 ng/mL) as an internal standard. Proteins were precipitated with methanol:acetone 1:1 (*v*/*v*), sonicated, and vortexed. After centrifugation, the supernatant was transferred into new tubes and dried. For UPLC–LCMS/MS analysis, the residue was redissolved in methanol. Quantitative analysis of lysoGb3 was performed on a Triple Quadripole mass spectrometer coupled to an Acquity UPLC system (Waters^®^) and equipped with an Acquity BEH-C18 column. Elution was achieved by mobile phase A, consisting of 37% methanol, 63% water containing 1 mM ammonium formiate, and 0.1% formic acid, and mobile phase B, consisting of 100% methanol containing 1 mM ammonium formiate and 0.1% formic acid. A calibration curve was generated by a serial dilution of lysoGb3 (Matreya-LLC) in methanol, with concentrations ranging from 100 to 1.56 ng/mL.

### 2.4. Alpha-d-Galactopyranosidase Activity Analysis

Alpha-d-galactopyranosidase enzymatic activity was determined in isolated blood leukocytes using a fluorometric assay [14]. Hexosaminidase activity was also determined as an enzyme control to confirm leukocyte integrity. The residual enzymatic activity (REA) is defined as the ratio of enzyme activity measured in a sample to the activity measured with a control.

### 2.5. Data Analysis

Spearman correlation analysis was used to explore associations and covariations. Euclidean distance was used as a similarity measure in the clustering analysis. False discovery rate correction was performed using the Benjamini–Hochberg–Yekutieli method, and 0.05 was considered as the statistical significance cut-off. Firstly, hierarchical cluster analysis was applied to the dataset to get an overview of the clustering trends of samples with similar profiles of variable intensity. Furthermore, multivariate data analysis and modeling were performed on log-transformed and unit-variance scaled data. Principal component analysis (PCA) was used as an unsupervised method. PCA was firstly applied to get an overview of the data and identify potential severe outliers which are defined as observations whose scores mapped outside the Hotelling’s T2 ellipse (confidence interval = 0.95) in a cross-validated seven-component model. For univariate analysis, a *t*-test was used for binary comparisons. Mann–Whitney test and Fisher’s exact test were used as non-parametric tests. The fraction of explained variability was measured as the sum of squares explained (SSE) and was determined using analysis of variance (ANOVA). All data analyses and visualizations were performed using R software (R Core Team, Vienna, Austria) [45]. The full data matrix is presented in Supplementary Table S1. 

## 3. Results

**Figure 2:**
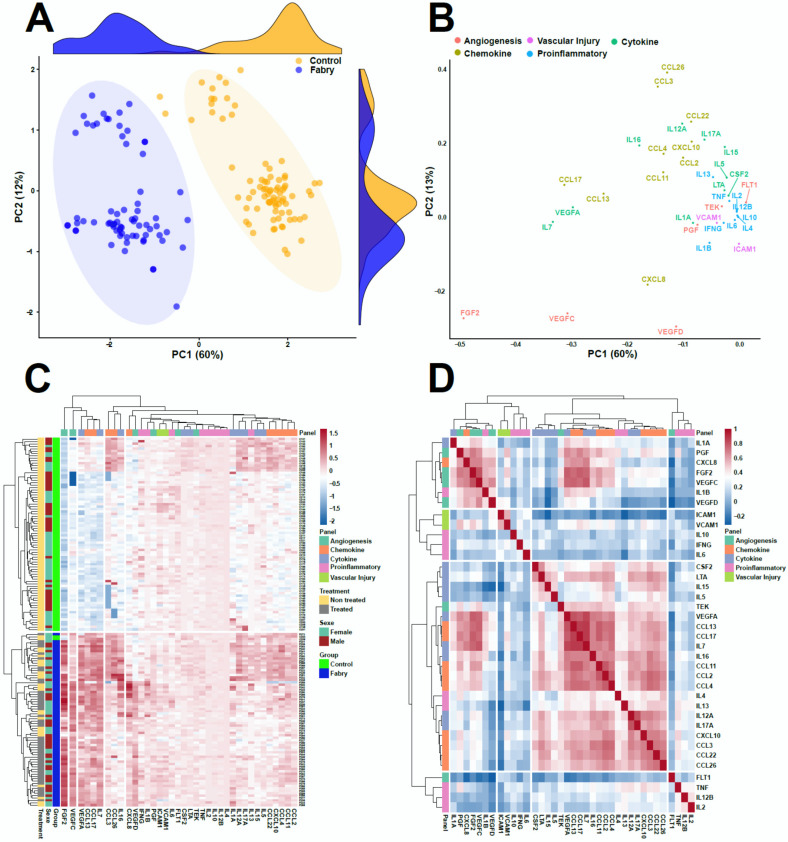
(**A**) Principal component analysis (PCA) score plot showing a clear separation between controls and Fabry samples. (**B**) PCA loadings showing the underlying proteins that drive this separation, mainly FGF2, IL-7, and VEGF. (**C**) Hierarchical cluster analysis of plasma samples based on protein levels. Classes are represented along the *y*-axis. The color code was used to represent log-scaled intensities of the proteins, showing the relative abundance of the protein according to the groups. The figure shows clear clustering. (**D**) Correlation heatmap between the assessed proteins and their hierarchical cluster analysis highlighting four main clusters. Low correlation is shown in blue and high correlation is shown in red.

**Figure 3:**
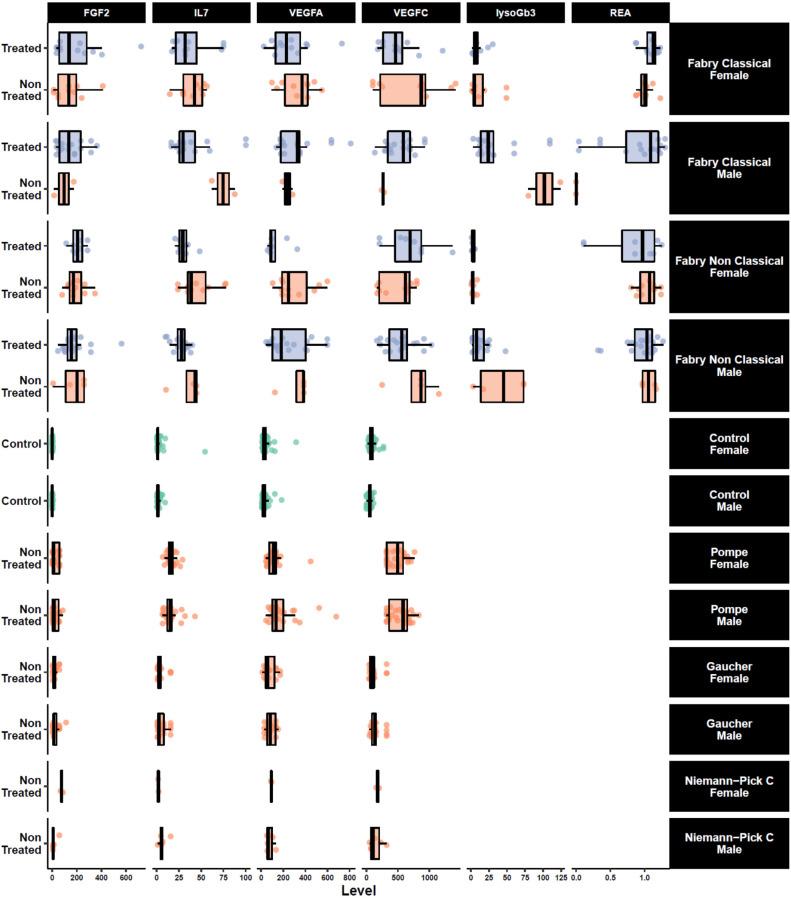
Boxplots of selected top significant proteins between sex-related controls and Fabry samples and their expression in phenotype-, sex-, and treatment-related Fabry, along with three other lysosomal diseases; Gaucher, Niemann–Pick Type C, and Pompe. FGF2; fibroblast growth factor 2, IL7; interleukin 7, VEGFA; vascular endothelial growth factor A, VEGFC; vascular endothelial growth factor C, REA; residual enzymatic activity, lysoGb3; globotriaosylsphingosine.

**Figure 4:**
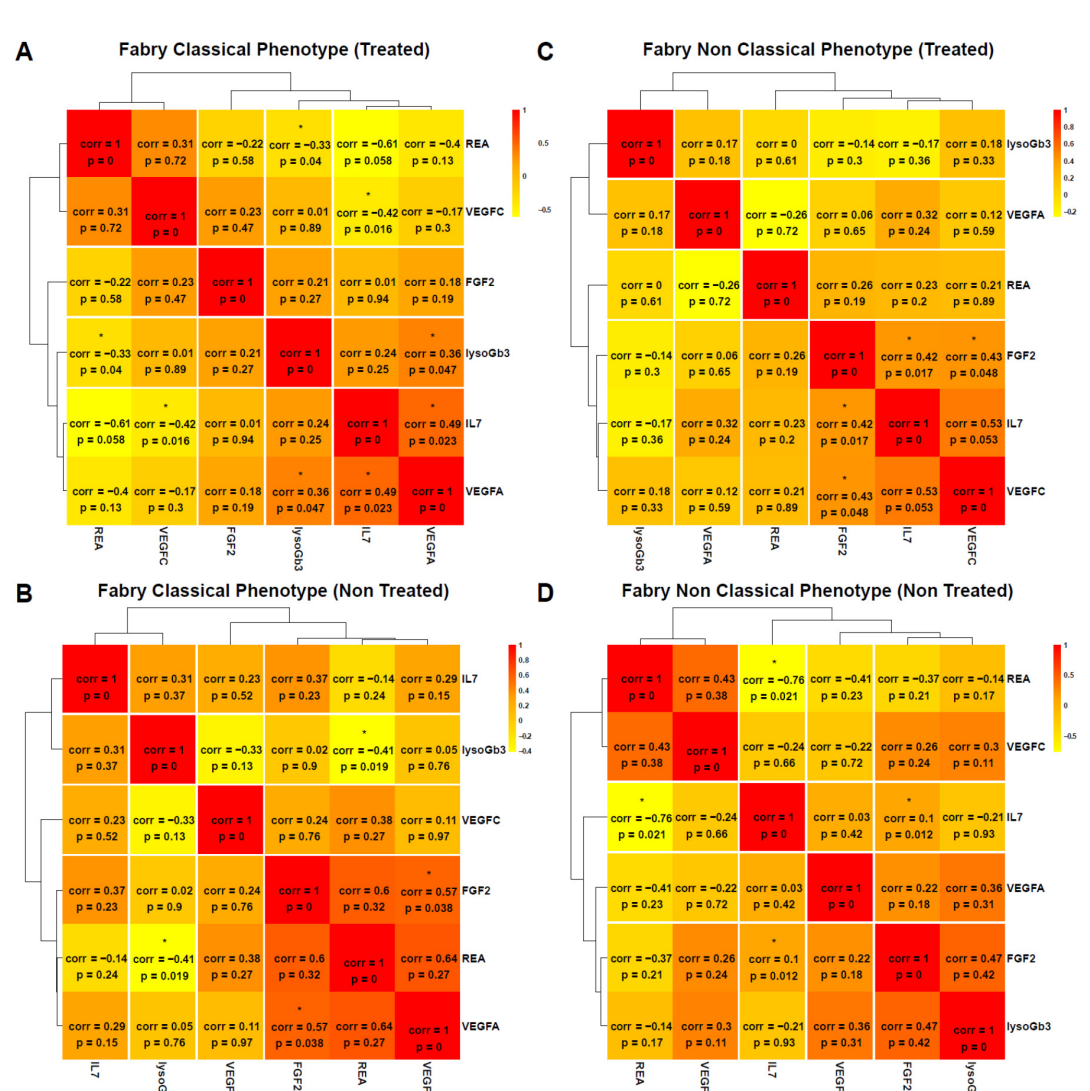
Correlation between LysoGb3, residual enzyme activity (REA), FGF2, IL-7, VEGFA, and VEGF across different Fabry subgroups related to treatment status and phenotype. (**A**) Correlated matrix for treated Fabry samples with classical phenotype. Significant correlations: REA vs. lysoGb3 (Corr = −0.33, *p* = 0.04); IL-7 vs. VEGFC (Corr = −0.42, *p* = 0.01); VEGFA vs. lysoGb3 (Corr = 0.36, *p* = 0.04); IL-7 vs. VEGFA (Corr = 0.49, *p* = 0.2). (**B**) Correlated matrix for non-treated Fabry samples with classical phenotype. Significant correlations: REA vs. lysoGb3 (Corr = −0.41, *p* = 0.01); FGF2 vs. VEGFA (Corr = 0.57, *p* = 0.03). (**C**) Correlated matrix for treated Fabry samples with non-classical phenotype. Significant correlations: FGF2 vs. VEGFC (Corr = 0.43, *p* = 0.04); FGF2 vs. IL-7 (Corr = 0.42, *p* = 0.01). (**D**) Correlated matrix for non-treated Fabry samples with non-classical phenotype. Significant correlations: IL-7 vs. REA (Corr = −0.76, *p* = 0.02); FGF2 vs. IL-7 (Corr = 0.1, *p* = 0.01). Corr = Spearman correlation, * = significant. FGF2; fibroblast growth factor 2, IL7; interleukin 7, VEGFA; vascular endothelial growth factor A, VEGFC; vascular endothelial growth factor C, REA; residual enzymatic activity, lysoGb3; globotriaosylsphingosine.

**Figure 5:**
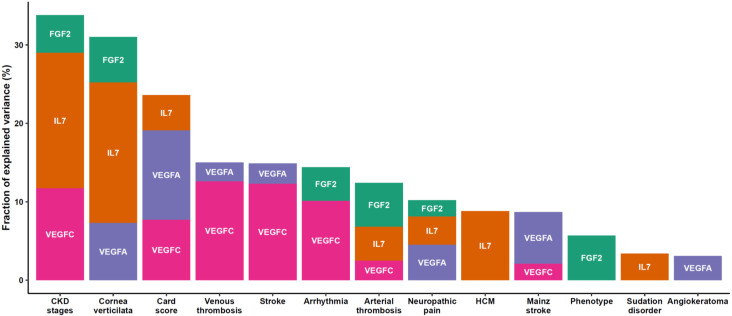
Barplot of variance explanation fraction of FGF2, IL-7, VEGFA, VEGFC, and related clinical characteristics. Colors are related to each protein. CKD; chronic kidney disease. HCM; hypertrophic cardiomyopathy. FGF2; fibroblast growth factor 2, IL7; interleukin 7, VEGFA; vascular endothelial growth factor A, VEGFC; vascular endothelial growth factor C.

To explore the data holistically, we applied a non-supervised method which is principal component analysis (PCA) to analyze the underlying differential biological profiles. A three-component PCA model accounting for 72% of the total variance was built. A PCA score plot was used to track clustering and potential outliers within the data. The PCA score plot in Figure 2A shows a spontaneous and a clear separation between Fabry and control samples. The loading plot presented in Figure 2B clearly shows the highly correlation of FGF2 with the control group which is in the same position in the loadings plot as the control samples in the scores plot. To intuitively explore the relationship between the samples and the underlying protein patterns, we performed a hierarchical cluster analysis based on correlation analysis and Euclidean distance as similarity metrics. The results shown in Figure 2C illustrate the clear separation and tight grouping of all samples belonging to their respective groups. The dendrogram structure highlights two main clusters of variable intensities represented by its two longest branches (maximum dissimilarity according to the Euclidean distance). The dendrogram structure of proteins highlights two main clusters that define main differential patterns according to the heatmap color gradient. This confirms the proteins yielded by the differential analysis that showed FGF2 as the main driver of this separation among the other cytokines and angiogenesis factors (IL-7 and VEGF family). Furthermore, we performed correlation analysis between the assessed proteins to track coexpressed modules that drive the underlying biological patterns. The results are shown in Figure 2D. The figure shows a heatmap visualization of the Spearman correlation between the 40 assessed proteins and shows clear coexpression patterns with highly differential proteins belong to the same cluster including other cytokines. Interestingly, the clusters underline the biological similarity of the different proteins. The largest one includes 17 proteins (CSF2, LTA, IL-15, IL-5, IL-13, IL-4, TEK, IL-16, CCL11, CCL2, CCL4, IL-12A, IL-17A, CXCL10, CCL3, CCL22, CCL26). This cluster contains mainly interleukins. The second cluster included 12 proteins (FGF2, IL-7, VEGF, VEGFA, VEGFC, VEGFD, IL-1B, IL-1A, PGF, CXCL8, CCL17, CCL13). This cluster is the most discriminant since it included all the differentially expressed proteins. Two other small clusters were also identified, one with five proteins (ICAM1, VCAM1, IL-10, IL-6, and IFNG) and one with four proteins (FLT1, TNF, IL-12B, and IL-2). Supplementary Table S3 presents the full correlation data. For deeper insight into the data, we performed a statistical analysis to assess the difference between FD patients and controls. The analysis yielded four proteins which were significantly expressed out of 40 (fold change >1.5 and adjusted *p*-value <0.05). These proteins include three angiogenesis proteins, FGF2, VEGFA, VEGFC, and one cytokine, IL-7 (Figure 3**)**. The results stayed consistent after subgrouping taking into account sex, treatment status, and Fabry phenotype. No differences were observed between treated and non-treated samples (Table 2). To evaluate the specificity of these variations, we also measured the concentration of these potential biomarkers in three other LDs (Gaucher, Pompe, and Niemann–Pick C). We observed a clear elevation of FGF2 and IL-7 in Fabry samples compared to all other LD samples. Statistical metrics and boxplots are shown in Table 2 and Figure 3, respectively. Full statistics are presented in Supplementary Table S4. We also assessed the correlation of these proteins with the widely used biomarker for Fabry disease, LysoGb3, and residual enzymatic activity (REA) in different subgroups of the Fabry samples regarding their treatment status and phenotype. The full results are presented in Figure 4, and related scatter plots are presented in Figures S1–S4 (Supplementary Materials). Overall, the results show few significant correlations; however, the correlations showed patterns related to phenotype and treatment subgroups. For correlations in treated Fabry samples with classical phenotype, four significant correlations were observed including REA vs. lysoGb3 (Corr = −0.33, *p* = 0.04), IL-7 vs. VEGFC (Corr = −0.42, *p* = 0.01), VEGFA vs. lysoGb3 (Corr = 0.36, *p* = 0.04), IL-7 vs. VEGFA (Corr = 0.49, *p* = 0.2). For non-treated Fabry, samples with classical phenotype yielded two significant correlations for REA vs. lysoGb3 (Corr = −0.41, *p* = 0.01), FGF2 vs. VEGFA (Corr = 0.57, *p* = 0.03). For treated Fabry samples with non-classical phenotype, the significant correlations were FGF2 vs. VEGFC (Corr = 0.43, *p* = 0.04), FGF2 vs. IL-7 (Corr = 0.42, *p* = 0.01). D). Finally, for non-treated Fabry samples with non-classical phenotype, the significant correlations were IL-7 vs. REA (Corr = −0.76, *p* = 0.02), FGF2 vs. IL-7 (Corr = 0.1, *p* = 0.01). The latter correlation was discarded due to it being very low. To evaluate the effect of these proteins on the main clinical parameters presented in Supplementary Table S1, we performed ANOVA analysis and extracted the explained variance of each protein. The results show that VEGFC is linked mainly to chronic kidney disease (CKD) stages, cardiac score, thrombosis, stroke, and arrhythmia, whereas VEGFA is related to cornea verticiltata, cardiac score, venous thrombosis, stroke, neuropathic pain, and solely to angiokeratoma. Furthermore, IL-7 is linked to CKD stages, cornea verticilata, cardiac score, arterial thrombosis, neuropathic pain, and solely to hypertrophic cardiomyopathy (HCM) and sudation disorder. FGF2 is related to CKD stages, cornea verticilata, arrhythmia, arterial thrombosis, neuropathic pain, and solely to phenotype (Figure 5**)**. The ANOVA results are presented in Supplementary Table S5.

## 4. Discussion

Fabry disease is primarily a vascular and inflammatory pathology [1]. Thus, 40 proteins, involved in inflammatory and angiogenesis processes, were measured in plasma samples from FD patients and healthy controls. This study revealed four proteins with highly significant differential expression levels between the studied groups and sex and phenotype-related subgroups. This proteomic signature includes three angiogenesis-related proteins (FGF2, VEGFA, VEGFC) and one cytokine (IL-7). Importantly, these concentrations are significantly increased in female and male patients with similar range of values (Figure 4).

Furthermore, we assessed the correlation between these four proteins and the conventional biomarker LysoGb3 and residual enzymatic activity (REA). Given the phenotype differences in FD patients, we sub-stratified the correlation analysis according to their phenotype and treatment status. The analysis yielded different patterns. In classical phenotype, LysoGb3 was negatively correlated with REA in both treated and non-treated samples (Corr = −0.33, *p* = 0.04) and (Corr = −0.41, *p* = 0.01), respectively. However, it was positively correlated with VEGFA in non-treated classical phenotype (Corr = 0.36, *p* = 0.04) only. No significant correlation was observed in non-classical phenotype for lysoGb3. However, for REA, we observed a high correlation with IL-7 non-treated samples with non-classical phenotype (Corr = −0.76, *p* = 0.02). This phenotype-related pattern stresses the need for deeper patient stratification in FD. The absence of correlation between FGF2 and IL-7 with LysoGb3 emphasis the potential orthogonal biological information that is carried by these proteins compared to LysoGb3. These difference exhibit the potential of this signature in stratifying and monitoring Fabry patients. 

The association analysis between the observed changes in these proteins and the clinical parameters showed interesting patterns. Overall, main FD features including hypertrophic cardiomyopathy, venous thrombosis, arterial thrombosis, and stroke, cornea verticilata, and renal (chronic kidney disease stage) events are partly explained by FGF2, IL-7, VEGFA, and VEGFC plasma levels. Of note, FGF2, IL-7 and VEGFA explained solely a small variance fraction of phenotype, sudation disorder, and angiokeratoma, respectively (Figure 5).

It is now recognized that female FD patients present with a wide range of clinical symptoms with variable severity and onset. Forty percent of female Fabry patients have normal leukocyte alpha-galactosidase A activity. Furthermore, several female patients who are affected with a sequence variant leading to a prominent cardiac variant, i.e., N215S have normal LysoGb3 blood level [46]. Thus, our findings may represent an important achievement in the characterization of FD in these patients. 

The specificity of these putative biomarkers was also assessed in adult patients with three other LDs (Gaucher, Pompe, and Niemann–Pick C). The results showed a clear elevation of FGF2 and IL-7 in Fabry disease compared to all other LDs. These changes seem to be specific to metabolic impairments inherent to Fabry disease and might not be related to common alterations to LDs. 

The fibroblast growth factor 2 (FGF2), also named bFGF for basic FGF, belongs to the FGF family which counts 22 members. FGF2 regulates cell differentiation and tissue growth during developmental stages and cellular life, and it is expressed in numerous tissues (brain, muscles, bones). FGF2 binds to fibroblast growth factor receptor (FGFR) proteins and exerts several biological functions such as strong angiogenic effects, cell growth, and tissue repair [47]. FGF2 homeostasis is disrupted in several diseases including cancer and cardiovascular diseases. Lee et al. reported an upregulation of Transforming growth factor beta 1 (TGF-β1), VEGF, VEGFR2, FGF2, and P-p38 expression in the Fabry mouse kidney. Furthermore, Gb3 treatment of cultured endothelial cells resulted in the overexpression of these proteins [48]. These results are in line with the increase of FGF2 plasma concentration in Fabry compared to control reported in the present study. Moreover, FGF2 overexpression promotes tumor growth and progression. It was reported that FGF2 inhibition or FGF2/FGFR signaling pathway may reduce tumor progression [49,50]. FGF2 upregulation may underlie endothelial dysfunction [51]. Cancer prevalence in Fabry disease is debated [52,53]. Future studies of tumors arising in Fabry patients may help to shed light on a potential involvement of FGF2 deregulation. Lowering FGF2 plasma concentration in Fabry patients could prove to be an interesting therapeutic approach in Fabry treatment. The possible use of FGF2/FGFR inhibitors as combinatory therapy with specific treatment should be sustained by complementary studies to confirm the correlation of FGF2 concentration with symptomatology.

It was already demonstrated that inflammation processes are activated in LDs [54]. The proposed mechanism relies on glycolipid accumulation that stimulates the activation of pathogenic inflammatory response via interactions between Toll-like receptors and Gb3 or its related metabolites. Inflammation is a major component of the Fabry disease pathogeny and the resulting tissues damages could be potentiated by those of autophagy disruption [55,56] and oxidative stress [41]. Heo et al. reported C3-mediated complement activation [38]; pro-inflammatory proteins, IL-6, and MCP-1 were also reported by Chen et al. as potential biomarkers in FD [57]. Yogasundaram et al. described systemic inflammatory patterns in FD patients associated with heart failure with preserved ejection fraction. The authors reported associations of kidney disease, left-ventricular hypertrophy, and myocardial fibrosis, with higher levels of inflammation and biomarkers of adverse cardiac remodeling [35]. Other studies also comforted the role of cardiac markers such cardiac troponin T (TNT) and brain natriuretic peptide (BNP) in FD hypertrophic cardiomyopathy [58,59,60].

Interleukin-7 is a mandatory cytokine for T-cell survival and homeostasis. Thus, IL-7 deficiency causes severe lymphopenia. The IL-7 pathway interacts closely with Vps34, the class III PI3K involved in autophagy regulation [61]. Increased IL-7 production plays a role in the predisposition to autoimmunity and inflammation [62]. The increase in IL-7 level, observed in this study, may contribute to autophagy impairment [56] and inflammation [54] observed in Fabry disease. 

As illustrated in Figure 4 and Table 2, there is no significant difference in FGF2 and IL-7 concentrations between treated and non-treated Fabry patients. However, the relatively small number of included Fabry patients treated versus untreated patients, the heterogeneity of the given specific treatments, and the variability in the duration of treatment do not allow the accurate assessment of the treatment effect on FGF2 and IL-7 plasma concentrations. It can be assumed that this effect might depend on the treatment type and the pathways involved for its processing and action. For instance, it was recently reported that neutralizing antibodies toward exogenous enzyme may inhibit endothelial internalization and reduce enzyme activity [44,63]. Thus, plasma variations of a potential endothelial biomarker may depend on the expression of neutralizing antibodies in patients treated with ERT.

Sample size is an obvious limitation when generalizing the findings of this work. Hence, further studies, on a larger cohort, are needed to explore the potential predictive power of this proteomic signature in Fabry patients as a diagnosis tool for FD, especially in mild forms and heterozygous women. Its potential as a surrogate biomarker signature for treatment monitoring using different specific treatment, agalsidase α, agalsidase β, or Migalastat, should also be tested on larger cohorts.

## 5. Conclusions

In this study, a plasma-based targeted proteomic approach revealed significant protein impairments in patients with Fabry disease. The identification of pathological signatures may provide a better understanding of the pathophysiological mechanism underlying these diseases and, thus, allow therapeutic innovation in such rare diseases. Further studies, including a larger cohort, are needed to define the predictive power of this signature and its potential use as FD biomarkers for diagnosis and treatment monitoring. 

## Figures and Tables

**Table 1:** Clinical characteristics of the cohort.

	Summary	*n*	Fabry Classical *n* = 34 (14%) ^1^	Fabry Non-Classical *n* = 35 (14%) ^1^	Control *n* = 83 (33%) ^1^	Pompe *n* = 59 (24%) ^1^	Gaucher *n* = 30 (12%) ^1^	Niemann–Pick C *n* = 8 (3.2%) ^1^	*p*-Value ^2^
**Age (Years)**	mean (SD)	248	44 (15)	48 (15)	42 (13)	54 (13)	41 (18)	45 (13)	0.3
**Sex**		249							0.5
*Female*	n/N (%)		20/34 (59%)	15/35 (43%)	44/83 (53%)	30/59 (51%)	14/30 (47%)	2/8 (25%)	
*Male*	n/N (%)		14/34 (41%)	20/35 (57%)	39/83 (47%)	29/59 (49%)	16/30 (53%)	6/8 (75%)	
**BMI (kg/m^2^)**	mean (SD)	56	25 (7)	29 (16)					0.2
**Cornea verticilata**	n/N (%)	57	26/26 (100%)	3/31 (9.7%)					**<0.001**
**Hypertrophic cardiomyopathy**	n/N (%)	67	14/33 (42%)	19/34 (56%)					0.3
**Angiokeratoma**	n/N (%)	66	20/32 (62%)	12/34 (35%)					0.048
**Arterial thrombosis**	n/N (%)	65	1/31 (3.2%)	2/34 (5.9%)					>0.9
**Venous thrombosis**	n/N (%)	66	6/32 (19%)	1/34 (2.9%)					0.051
**Arrhythmia**	n/N (%)	69	19/34 (56%)	14/35 (40%)					0.2
**Stroke**	n/N (%)	67	5/32 (16%)	5/35 (14%)					>0.9
**Neuropathic pain**	n/N (%)	66	31/31 (100%)	15/35 (43%)					**<0.001**
**Sudation disorder**	n/N (%)	69	24/34 (71%)	13/35 (37%)					**0.008**
**Kidney Transplant**	n/N (%)	69	3/34 (8.8%)	0/35 (0%)					0.11
**Dialysis**	n/N (%)	68	30/34 (88%)	34/34 (100%)					0.11
**CKD stages**		66							0.2
*0*	n/N (%)		18/33 (55%)	18/33 (55%)					
*1*	n/N (%)		2/33 (6.1%)	3/33 (9.1%)					
*2*	n/N (%)		6/33 (18%)	9/33 (27%)					
*3*	n/N (%)		1/33 (3.0%)	3/33 (9.1%)					
*4*	n/N (%)		2/33 (6.1%)	0/33 (0%)					
*5*	n/N (%)		4/33 (12%)	0/33 (0%)					
**Treatment**		69							>0.9
*Non-Treated*	n/N (%)		11/34 (32%)	12/35 (34%)					
*Treated*	n/N (%)		23/34 (68%)	23/35 (66%)					
**Therapy**		69							0.3
*Agalsidase α*	n/N (%)		9/34 (26%)	3/35 (8.6%)					
*Agalsidase α/Agalsidase β*	n/N (%)		4/34 (12%)	6/35 (17%)					
*Agalsidase α/Agalsidase β/Miglastat*	n/N (%)		0/34 (0%)	1/35 (2.9%)					
*Agalsidase α/Miglastat*	n/N (%)		0/34 (0%)	1/35 (2.9%)					
*Agalsidase β*	n/N (%)		10/34 (29%)	11/35 (31%)					
*Miglastat*	n/N (%)		0/34 (0%)	1/35 (2.9%)					
*Non-Treated*	n/N (%)		11/34 (32%)	12/35 (34%)					
**Treatment Duration (Years)**	mean (SD)	45	6.8 (4.8)	6.0 (5.2)					0.6
**Variant**		63							**0.011**
*Missense*	n/N (%)		12/29 (41%)	25/34 (74%)					
*MTP*	n/N (%)		17/29 (59%)	9/34 (26%)					
**Neutralizing Antibody (Positive)**	n/N (%)	69	30/34 (88%)	33/35 (94%)					0.4
**lysoGb3 (ng/mL)**	mean (SD)	63	22 (31)	10 (15)					**0.011**
**Residual Enzymatic Activity (%)**	mean (SD)	57	0.94 (0.37)	0.98 (0.29)					>0.9
**MDRD (mL/min)**	mean (SD)	63	85 (41)	102 (30)					0.14

^1^ Statistics are presented as mean (SD); n/N (%). ^2^ Statistical tests performed between classical and non-classical. Fabry: Mann–Whitney test for continuous variables; Fisher’s exact test for categorical variables. BMI; Body Mass Index, CKD; Chronic Kidney Disease, MTP; Mutations leading to a truncated protein (deletion, frameshift, or non-sense mutations), MDRD; Modification of Diet in Renal Disease.

**Table 2:** Statistical metrics of the most differentially expressed proteins.

Protein	Comparison	Log Fold Change	*p*-Value
FGF2	Fabry Non-Treated vs. Control	2.30	7.49 × 10^−26^
FGF2	Fabry Treated vs. Control	2.22	1.72 × 10^−23^
FGF2	Fabry Non-Treated vs. Gaucher	−1.33	1.87 × 10^−6^
FGF2	Fabry Treated vs. Gaucher	−1.24	7.46 × 10^−6^
FGF2	Fabry Treated Classic Female vs. Control Female	2.21	2.81 × 10^−17^
FGF2	Fabry Non-Treated Non-Classic Female vs. Control Female	2.26	1.45 × 10^−15^
FGF2	Fabry Treated Non-Classic Female vs. Control Female	2.24	3.20 × 10^−11^
FGF2	Fabry Non-Treated Classic Male vs. Control Female	2.00	1.45 × 10^−3^
FGF2	Fabry Non-Treated vs. Fabry Treated	−0.09	8.63 × 10^−1^
IL-7	Fabry Treated vs. Pompe	−1.48	4.47 × 10^−11^
IL-7	Fabry Non-Treated vs. Pompe	−1.29	5.55 × 10^−9^
IL-7	Fabry Treated vs. Niemann Pick C	−1.80	8.17 × 10^−3^
IL-7	Fabry Non-Treated vs. Niemann Pick C	−1.60	2.12 × 10^−2^
IL-7	Fabry Treated Classic Female vs. Control Female	2.10	1.73 × 10^−16^
IL-7	Fabry Non-Treated Classic Female vs. Control Female	1.94	2.21 × 10^−12^
IL-7	Fabry Non-Treated Non-Classic Female vs. Control Female	1.89	7.67 × 10^−12^
IL-7	Fabry Treated Non-Classic Female vs. Control Female	2.13	9.92 × 10^−11^
IL-7	Fabry Non-Treated vs. Fabry Treated	0.19	8.63 × 10^−1^
VEGFA	Fabry Non-Treated vs. Control	1.82	6.38 × 10^−15^
VEGFA	Fabry Treated vs. Control	1.85	9.63 × 10^−15^
VEGFA	Fabry Treated vs. Pompe	−1.49	4.19 × 10^−9^
VEGFA	Fabry Non-Treated vs. Pompe	−1.47	5.31 × 10^−9^
VEGFA	Fabry Treated vs. Niemann Pick C	−1.01	3.20 × 10^−1^
VEGFA	Fabry Treated Classic Female vs. Control Female	1.75	6.00 × 10^−10^
VEGFA	Fabry Non-Treated Non-Classic Female vs. Control Female	1.85	1.66 × 10^−9^
VEGFA	Fabry Non-Treated Classic Female vs. Control Female	1.79	6.00 × 10^−9^
VEGFA	Fabry Treated Non-Classic Female vs. Control Female	2.02	5.14 × 10^−8^
VEGFA	Fabry Non-Treated Classic Male vs. Control Male	1.62	4.92 × 10^−2^
VEGFA	Fabry Non-Treated vs. Fabry Treated	0.02	9.71 × 10^−1^
VEGFC	Fabry Treated vs. Control	1.82	1.69 × 10^−15^
VEGFC	Fabry Treated vs. Pompe	−1.71	5.96 × 10^−12^
VEGFC	Fabry Non-Treated vs. Pompe	−1.45	2.25 × 10^−9^
VEGFC	Fabry Treated Classic Female vs. Control Female	2.02	8.04 × 10^−14^
VEGFC	Fabry Non-Treated Non-Classic Female vs. Control Female	1.71	3.71 × 10^−9^
VEGFC	Fabry Non-Treated vs. Fabry Treated	0.26	8.63 × 10^−1^

FGF2; fibroblast growth factor 2, IL7; interleukin 7, VEGFA; vascular endothelial growth factor A (VEGFA), vascular endothelial growth factor C (VEGFC).

## Supplementary Materials

The following are available online at https://www.mdpi.com/2077-0383/9/5/1325/s1: Figure S1: Scatter plots and correlation in treated Fabry samples with classical phenotype, Figure S2: Scatter plots and correlation in non-treated Fabry samples with classical phenotype, Figure S3: Scatter plots and correlation in treated Fabry samples with non-classical phenotype, Figure S4: Scatter plots and correlation in non-treated Fabry samples with non-classical phenotype, Table S1: Clinical and proteomic data, Table S2: Summary statistics of the clinical characteristic of the cohort, Table S3: Correlation results of the proteomic data, Table S4: Results of differential analysis statistics, Table S5: Results of the ANOVA analysis.
