# Supplementary material for: A Proteomics-Based Analysis Reveals Predictive Biological Patterns in Fabry Disease
Source: J Clin Med. 2020 May 2;9(5):1325. doi: 10.3390/jcm9051325 (PMC7290805; doi:10.3390/jcm9051325)
Supplement: Supplementary file 1 [file jcm-09-01325-s001.zip › Tebani_et_al_fabomics_supplementray_data.pdf]

Supplementary Materials for

A proteomics-based analysis reveals predictive biological patterns in Fabry disease

Abdellah Tebani [1], Wladimir Mauhin [2], Lenaig Abily-Donval [3,4], Céline Lesueur [1,4], Marc G. Berger [5,6], Yann Nadjar [7], Juliette Berger [5,6], Oliver Benveniste [8], Foudil Lamari [9], Pascal Laforêt [10], Esther Noel[11], Stéphane Marret [3,4], Olivier Lidove [2], Soumeya Bekri [1] and [4] *

[1] Department of Metabolic Biochemistry, Rouen University Hospital, Rouen, 76000, France
[2] Department of Internal Medicine, Groupe Hospitalier Diaconesses Croix Saint Simon, Paris, France & INSERM U 974, 75014 Paris, France
[3] Department of Neonatal Pediatrics, Intensive Care and Neuropediatrics, Rouen University Hospital, Rouen, 76000, France
[4] Normandie Univ, UNIROUEN, CHU Rouen, INSERM U1245, 76000 Rouen, France
[5] CHU Clermont-Ferrand, Hopital Estaing, CRB-Auvergne, 63003, Clermont-Ferrand, France.
[6] Université Clermont Auvergne, EA 7453 CHELTER, 63000 Clermont-Ferrand
[7] Neurology Department, Reference center for Lysosomal Diseases, Hôpital Pitié-Salpêtrière, Paris, France
[8] Department of Internal Medicine, Hôpital Pitié-Salpêtrière, Paris, France & INSERM U 974, 75014 Paris, France
[9] Department of Metabolic Biochemistry, Pitié-Salpêtrière Hospital, APHP-Sorbonne university, Paris, France
[10] Neurology Department, Hôpital Raymond-Poincaré, AP-HP, Garches, France
[11] Department of Internal Medicine, Centre Hospitalier Universitaire, Strasbourg, France.
* Correspondence: soumeya.bekri@chu-rouen.fr

Content

Figure S1. Scatter plots and correlation between LysoGb3, residual enzyme activity (REA), FGF2, IL7, VEGFA, and VEGF. Data from treated Fabry samples with classical phenotype. Significant correlations: REA .vs. lysoGb3 (Corr = -0.33, p = 0.04), IL7 .vs. VEGFC (Corr = -0.42, p = 0.01), VEGFA .vs. lysoGb3 (Corr = 0.36, p = 0.04), IL7 .vs. VEGFA (Corr = 0.36, p = 0.04).

Figure S2. Scatter plots and correlation between LysoGb3, residual enzyme activity (REA), FGF2, IL7, VEGFA, and VEGF. Data from non-treated Fabry samples with classical phenotype. Significant correlations: REA .vs. lysoGb3 (Corr = -0.41, p = 0.01), FGF2 .vs. VEGFA (Corr = 0.57, p = 0.03).

Figure S3. Scatter plots and correlation between LysoGb3, residual enzyme activity (REA), FGF2, IL7, VEGFA, and VEGF. Data from treated Fabry samples with non-classical phenotype. Significant correlations: FGF2 .vs. VEGFC (Corr = 0.42, p = 0.01), FGF2 .vs. IL7 (Corr = 0.10, p = 0.01).

Figure S4. Scatter plots and correlation between LysoGb3, residual enzyme activity (REA), FGF2, IL7, VEGFA, and VEGF. Data from non-treated Fabry samples with non-classical phenotype. Significant correlations: IL7 .vs. REA (Corr = -0.76, p = 0.02), FGF2 .vs. IL7 (Corr = 0.42, p = 0.01). corr = Spearman correlation.

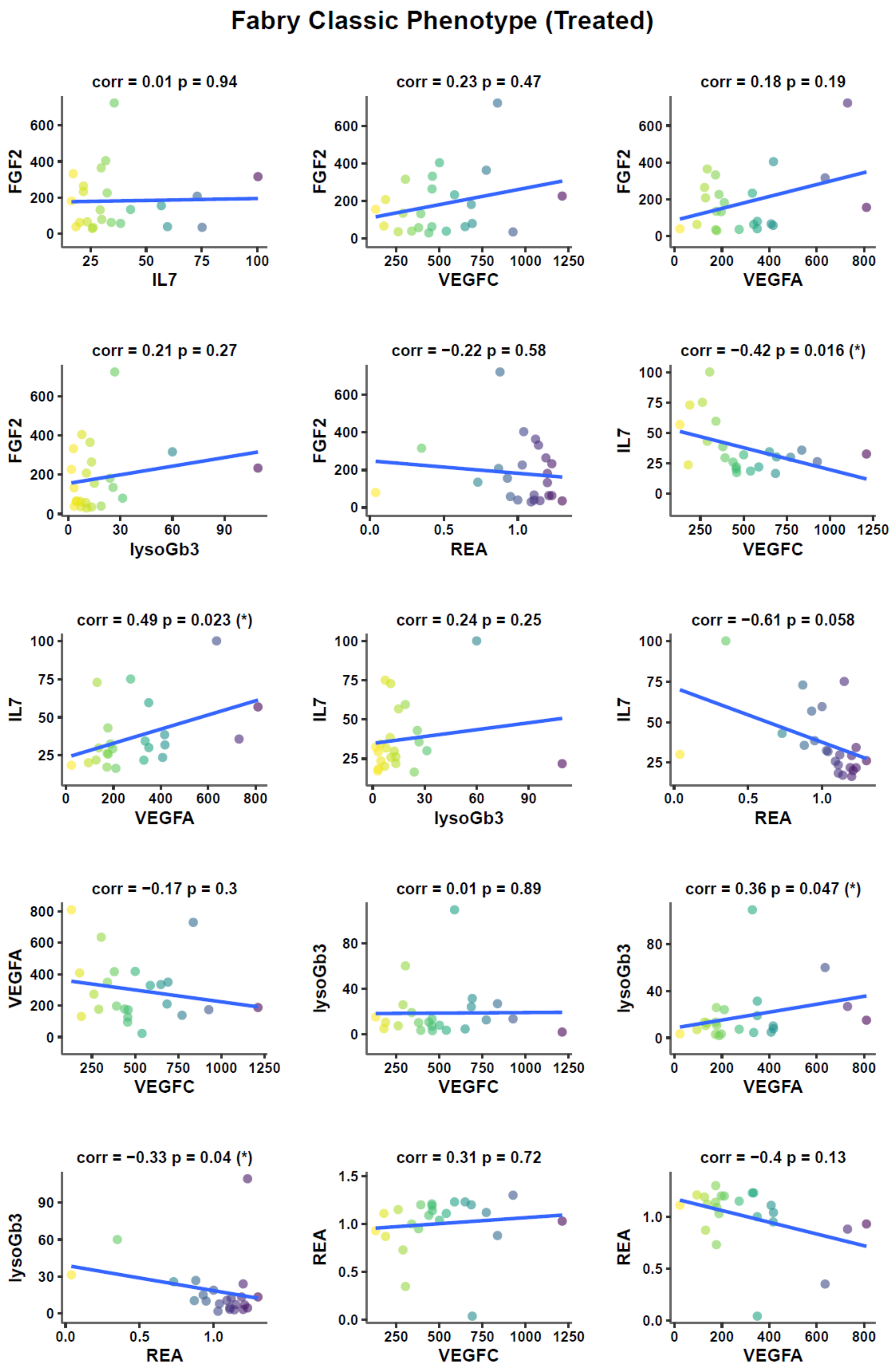

Figure S1. Scatter plots and correlation between LysoGb3, residual enzyme activity (REA), FGF2, IL7, VEGFA, and VEGF. Data from treated Fabry samples with classical phenotype. Significant correlations: REA .vs. lysoGb3 (Corr = -0.33, p = 0.04), IL7 .vs. VEGFC (Corr = -0.42, p = 0.01), VEGFA .vs. lysoGb3 (Corr = 0.36, p = 0.04), IL7 .vs. VEGFA (Corr = 0.49, p = 0.2).

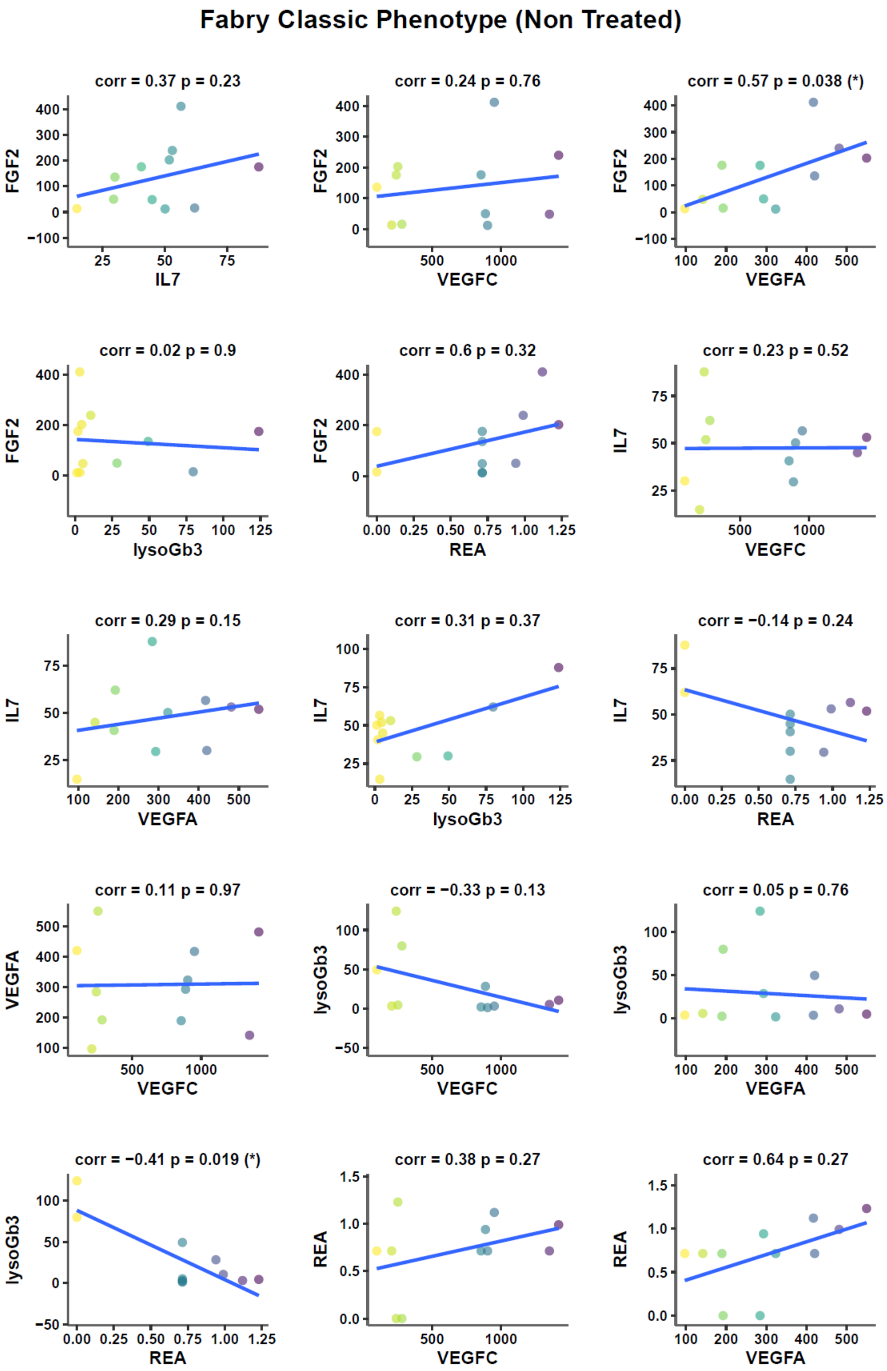

Figure S2. Scatter plots and correlation between LysoGb3, residual enzyme activity (REA), FGF2, IL7, VEGFA, and VEGF. Data from non-treated Fabry samples with classical phenotype. Significant correlations: REA .vs. lysoGb3 (Corr = -0.41, p = 0.01), FGF2 .vs. VEGFA (Corr = 0.57, p = 0.03).

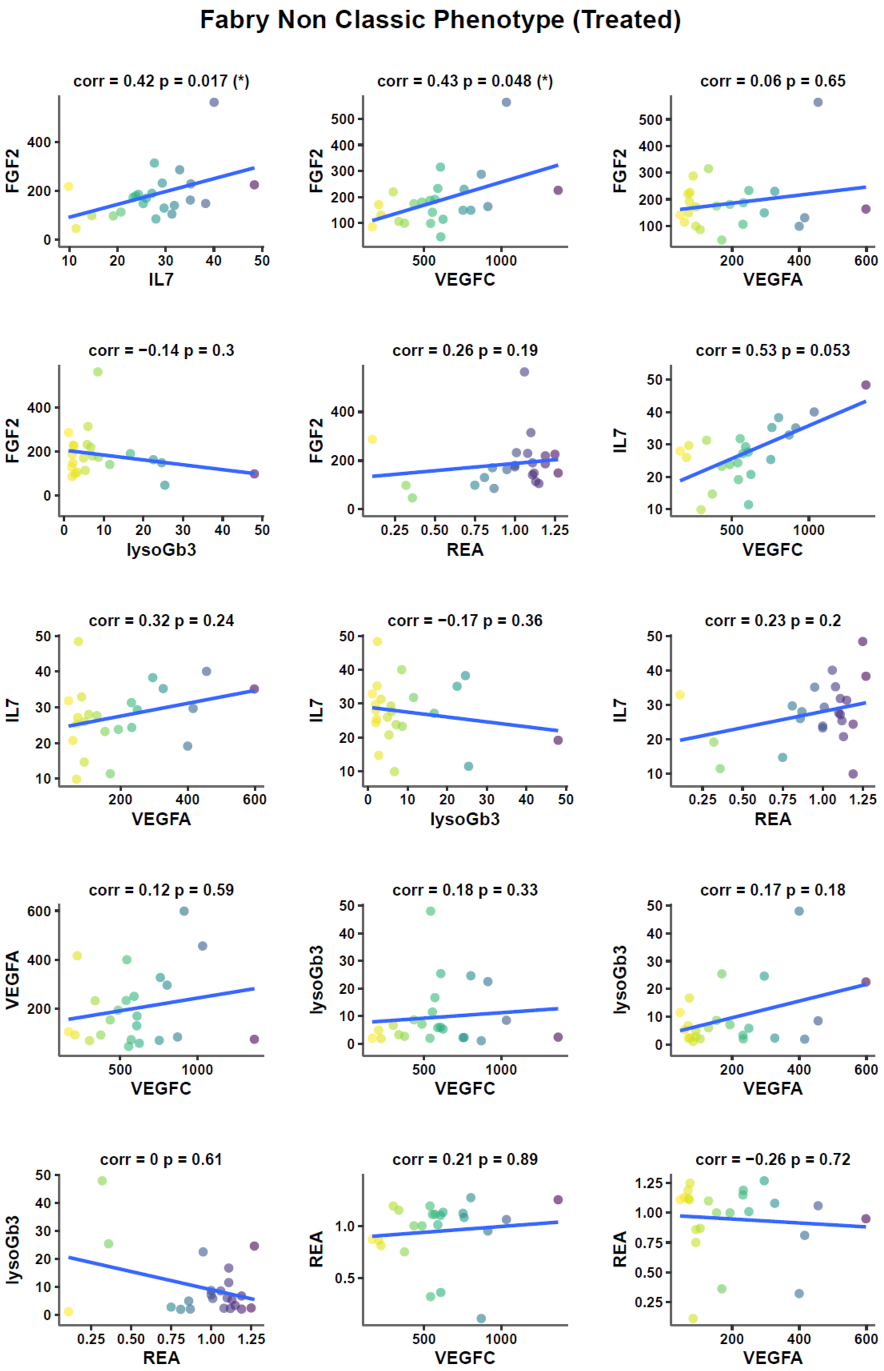

Figure S3. Scatter plots and correlation between LysoGb3, residual enzyme activity (REA), FGF2, IL7, VEGFA, and VEGF. Data from treated Fabry samples with non-classical phenotype. Significant correlations: FGF2 .vs. VEGFC (Corr = 0.43, p = 0.04), FGF2 .vs. IL7 (Corr = 0.42, p = 0.01).

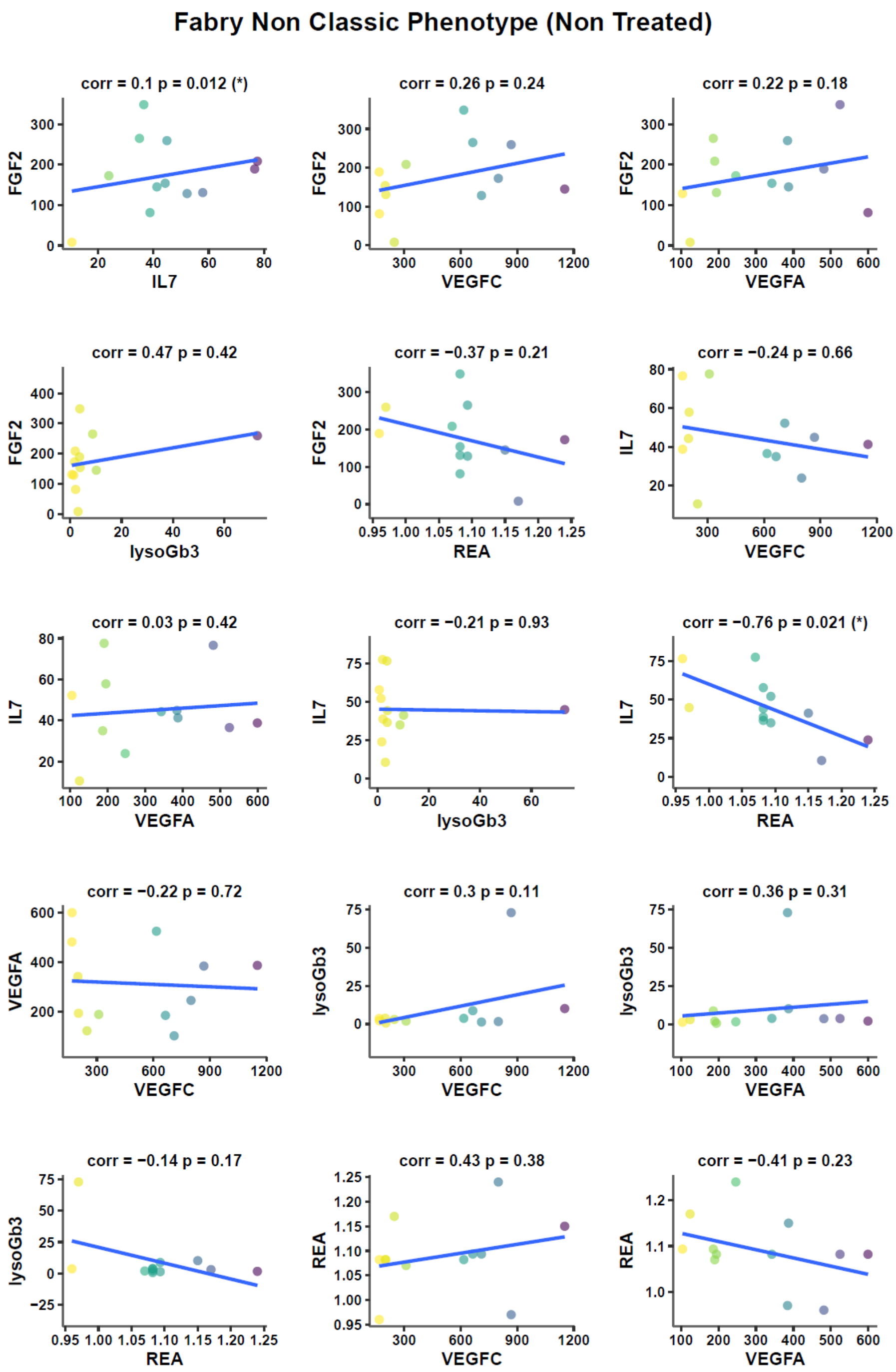

Figure S4. Scatter plots and correlation between LysoGb3, residual enzyme activity (REA), FGF2, IL7, VEGFA, and VEGF. Data from non-treated Fabry samples with non-classical phenotype. Significant correlations: IL7 .vs. REA (Corr = -0.76, p = 0.02), FGF2 .vs. IL7 (Corr = 0.1, p = 0.01). corr = Spearman correlation. Asterix * = Significant.
